# Tumor promoting effect of spheroids in an orthotopic prostate cancer mouse model

**DOI:** 10.1038/s41598-024-59052-0

**Published:** 2024-04-17

**Authors:** Julius Lars Daniel Bastian, Philip Zeuschner, Michael Stöckle, Kerstin Junker, Johannes Linxweiler

**Affiliations:** Department of Urology and Pediatric Urology, Saarland University, Kirrbergerstr. 100 Gebäude 6, 66424 Homburg, Germany

**Keywords:** Urological cancer, Prostate, Cancer microenvironment, Cancer models, Tumour heterogeneity, Experimental models of disease, Animal disease models, Cancer models, Prostate cancer, Cancer microenvironment, Cancer models

## Abstract

In this study, we aimed to establish a technique for intraprostatic implantation of prostate cancer (PCa) spheroids and to identify the impact of three-dimensional organization of PCa cells on tumor progression and metastasis in a representative in vivo model. 40,000 LNCaP cells were implanted into the prostate of immunodeficient SCID mice either as single cells (n = 8) or as preformed 3D spheroids (n = 8). For a follow up of 20 weeks, tumor growth was monitored by serum PSA and high-resolution 3D ultrasonography. Eventually, animals were sacrificed and autopsied. The organ dissects were analyzed for the presence of metastases by histology (H&E) and immunohistochemistry (AMACR, AR, Ki-67, CK5, CK8, E-Cadherin, Vimentin). Solid intraprostatic tumors developed in 50% of mice after spheroid implantation and in 50% of mice after implantation of a single cells. Primary tumors of LNCaP spheroids evolved earlier, exhibiting a shorter tumor doubling time whilst developing larger tumor volumes, which was reflected by a higher immunohistochemical expression of Ki-67 and AR, too. Spheroid tumors established lung and lymph node metastases in 75% of mice, in contrast to 50% of mice after single cell implantation. Our technique enables a variety of studies regarding the influence of the tumor microenvironment on PCa progression.

## Introduction

Prostate cancer (PCa) is one of the most common malignant diseases in men worldwide^1^. It primarily affects the elderly and is associated with high morbidity and mortality^2^. The relevance of PCa is expected to further increase in the future primarily because of demographic changes leading to an ageing society especially in industrialized countries, where PSA-screening is widely adopted^3^.

However, diagnostic and therapeutic options are limited due to a lack of understanding the distinct and heterogeneous biology of PCa^4^. Currently, there are only few preclinical models available to capture the complex tumor biology of PCa^5^. In recent years, three-dimensional cell culture models have gained increasing scientific interest^4^. Three-dimensional spheroid cultures are multicellular tumors that form a natural oxygen and nutrient gradient, thus recapitulating the natural tumor environment more accurately than conventional two-dimensional monolayer cell cultures^6–8^. However, the transfer of PCa spheroids into in-vivo models is rarely performed mainly due to the high technical demands of such an approach.

In current PCa in vivo studies, xenografts are typically implanted as conventional single-cell suspensions from monolayer cultures. However, this approach inadequately addresses the complex three-dimensional tumor microenvironment. While some studies on heterotopic injection of 3D cell clusters have been published^9–11^, orthotopic implantation techniques for preformed 3D cell culture systems for PCa have not been established yet. Previously, patient-derived xenografts (PDX) by means of intact tumor tissue samples or tissue-derived spheroid cultures have been successfully implanted orthotopically^12–15^. However, the orthotopic implantation of larger preformed 3D cell clusters with predefined number and composition of cells that mimic the tumor architecture more accurately is yet to be achieved. Thus, information on the potential effects of the three-dimensional structure of implanted cells on primary tumor growth and development of metastases in orthotopic in vivo models is currently lacking. The purpose of this work was to establish a preclinical in vivo model that enables a more precise representation of PCa biology by orthotopic implantation of preformed 3D spheroids and to assess if the three-dimensional organization of implanted PCa cells has an impact on primary tumor growth and the development of metastases. To achieve this, we first established a reliable method for generating three-dimensional PCa spheroids from LNCaP monolayer cells and assessing their morphology, proliferation and viability. Furthermore, a methodology for the intraprostatic implantation of preformed 3D LNCaP spheroids was established to ultimately compare local and systemic tumor progression of orthotopically implanted LNCaP spheroids versus conventional LNCaP single cell suspensions.

## Results

### LNCaP spheroid morphology and doubling time

**Figure 1:**
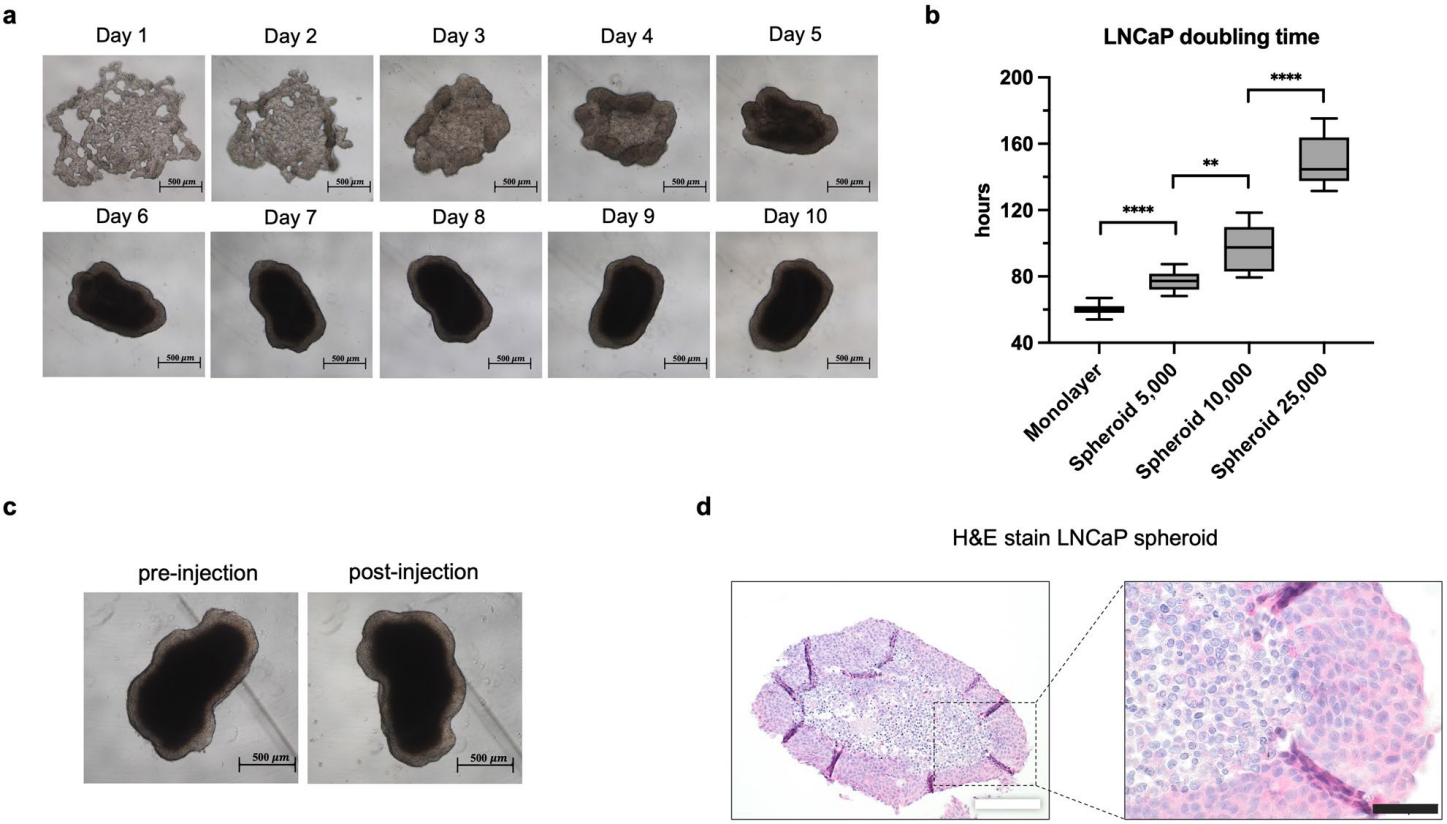
LNCaP spheroid morphology and doubling time. (**a**) Microscopic morphology of a spheroid within ten days after seeding in 96 well ULA plates (scale bar = 500 μm). (**b**) Doubling time (hours) in LNCaP monolayer and spheroids with increasing initial cell number (5,000; 10,000; 25,000). (Box plots min to max; n = 7; **** ≙ p < 0.0001; ** ≙ p < 0.01). (**c**) Microscopic integrity of a spheroid pre- and post-injection with a 20 G needle (scale bar = 500 μm). (**d**) Hematoxylin and Eosin stain of a 6-days maturated LNCaP spheroid from initially 25,000 seeded cells (white scale bar = 200 μm; black scale bar = 50 μm).

Small cell clusters formed one day after 25,000 LNCaP cells were seeded in ultra-low attachment 96-well plates. The cell clusters gradually aggregated over the course of the next two days to create a three-dimensional spheroid structure. The spheroids consolidated to a mean size of 1000 μm after ten days (Fig. 1a).

The doubling time of LNCaP monolayers was 60 h. However, the average generation time in spheroids significantly increased with a higher initial number of cells (Fig. 1b). Doubling time of spheroids with an initial cell number of 5,000 LNCaP was 77 h in contrast to 97 h of spheroids with an initial cell number of 10,000 (p < 0.01). The largest spheroids, which initially developed from 25,000 cells, exhibited the longest generation time of 150 h, which significantly differed from spheroids developed from 10,000 cells (p < 0.0001).

In preparation for the planned orthotopic in-vivo implantation, we assessed the integrity of preformed LNCaP spheroids before and after aspiration in and injection through a 20 G needle. Six-day matured spheroids initially cultivated from 25,000 cells maintained their morphology microscopically and histologically when aspirated and injected with a 20 G needle, unlike spheroids with higher cell numbers (Fig. 1c,d).

### Cell count and viability in LNCaP spheroids

**Figure 2:**
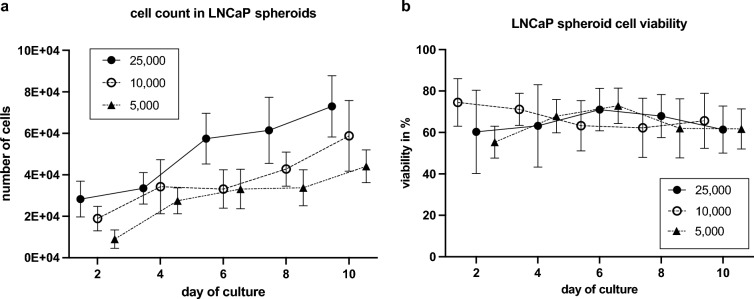
Cell count and viability of LNCaP spheroids. (**a**) Cell count in LNCaP spheroids with different initial cell numbers (5,000; 10,000; 25,000) within ten days (mean with SD; n = 7). (**b**) Cell viability in LNCaP spheroids with different initial cell numbers (5,000; 10,000; 25,000) within ten days (mean with SD; n = 7).

LNCaP spheroids showed steady growth over a period of ten days. A few spheroids experienced a decrease in total cell count two days after initial cultivation. All spheroids reached their maximum total cell count after ten days. The final number of LNCaP cells increased depending on the initial cell count. Spheroids initiated with 25,000 cells had an average cell count of 77,000 after ten days, while those with 10,000 cells increased to 59,000 after ten days. The smallest spheroids starting with 5,000 LNCaP cells reached a total of 44,000 cells after 10 days (Fig. 2a).

Cell viability in all three spheroid groups ranged from 50 to 85% (Fig. 2b). Spheroids with an initial count of 25,000 and 5,000 cells achieved the highest viability (70%) within ten days. Spheroids with an initial count of 10,000 cells showed a slight decrease in viability, dropping from an average of 75–65%.

### Orthotopic tumor cell engraftment and development of metastases

In few cases, the use of a large injection cannula resulted in perforation of the prostatic capsule impeding safe implantation of the LNCaP spheroids in the left anterior prostate lobe. In these cases, injection was successfully performed in the contralateral (right) anterior prostate lobe.

Four of eight mice conventionally transplanted with LNCaP single cells from monolayer culture developed tumors. Lung and lymph node metastases emerged from two of these tumors. Four out of eight mice implanted with spheroids showed tumors, three of which resulted in lung and lymph node metastases (Table 1).

**Table 1:** Development of primary tumors, lung and lymph node metastases based on histological examination of the dissected organs after 20 weeks of follow up.

	Tumor	Lung metastases	Lymph node metastases
LNCaP monolayer (n = 8)	n = 4 (50%)	n = 2 (50%)	n = 2 (50%)
LNCaP spheroid (n = 8)	n = 4 (50%)	n = 3 (75%)	n = 3 (75%)

### Sonographic tumor volume and PSA measurements

**Figure 3:**
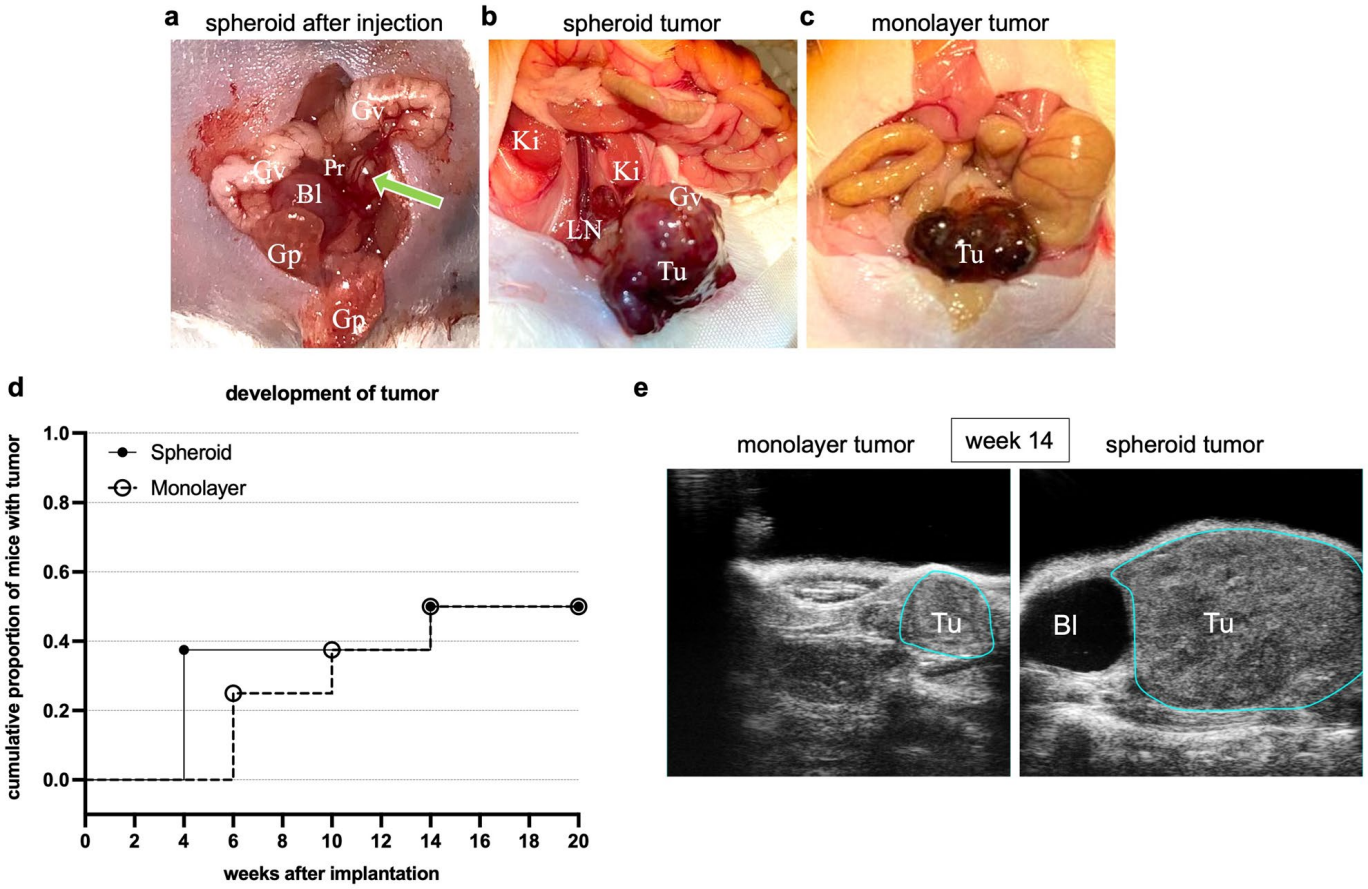
Orthotopic tumor growth. (**a**) Intraoperative situs after successful intraprostatic implantation of a LNCaP spheroid (marked with green arrow). (**b**,**c**) Intraoperative situs after 20 weeks with formation of a tumor (Tu) after LNCaP spheroid and LNCaP monolayer implantation (tenfold magnification). The glandulae vesiculosae (Gv), the glandulae praeputiales (Gp), the urinary bladder (Bl), the kidneys (Ki) and the left anterior prostate lobe (Pr) are visible. After twenty weeks, the implanted spheroid proliferated into a large tumor (Tu) with formation of lymph node metastases (LN). (**d**) Cumulative proportion of mice with tumor development within 20 weeks after implantation of a spheroid or single cell suspension from monolayer culture. (**e**) High-resolution ultrasonography images of a monolayer and spheroid tumor (Tu) at week 14. The tumors are marked with a blue line. Bl = bladder.

Successful intraprostatic implantation of a LNCaP spheroid was macroscopically confirmed (Fig. 3a). Using high-resolution small animal ultrasonography, the intestine, testicles, epididymis, vasa deferentia, and urinary bladder were identified and differentiated from the prostate. Ultrasound visualized local tumor growth in all tumor-bearing mice, as exemplary depicted in Fig. 3e. At autopsy, the sonographic tumor growth was macroscopically verified (Fig. 3b,c).

Three mice developed tumors four weeks after spheroid injection, while no tumors were detected in the monolayer injection group at this time point (Fig. 3d). A total of three monolayer tumors were not detectable until ten weeks after monolayer single cell injection (Fig. 3d). An additional mouse in each group developed a tumor at fourteen weeks (Fig. 3d).

**Figure 4:**
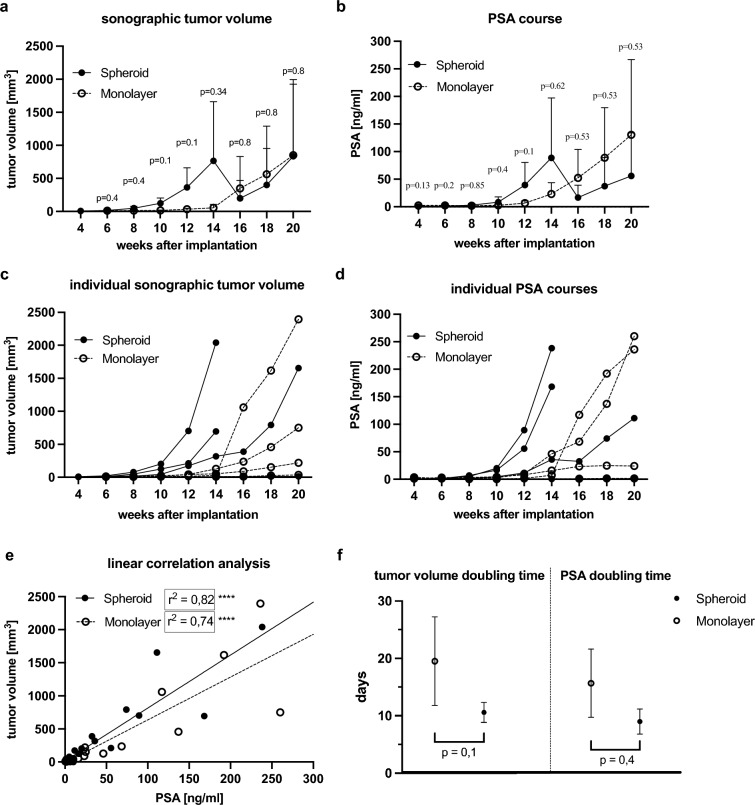
Development of primary tumor volume and serum PSA. (**a**) Sonographic tumor volume after orthotopic implantation of a spheroid (initial cell number 25,000) and injection of a cell-number matched single cell suspension from monolayer culture (40,000 cells per injection) in the span of 20 weeks (mean with SD). (**b**) PSA course of mice within 20 weeks after implantation (mean with SD). (**c**) Individual sonographic tumor volumes. (**d**) Individual PSA courses. (**e**) Linear correlation analysis of tumor volume and PSA in spheroid and monolayer tumors (**** ≙ p < 0.0001). (**f**) Tumor volume doubling times and PSA doubling times in days in spheroid and monolayer tumors (mean with SD).

At fourteen weeks, the mean primary tumor volume of LNCaP spheroid tumors (766 mm^3^) was nearly 14 times higher than that of LNCaP monolayer tumors (55 mm^3^, p = 0.34) (Fig. 4a,c). The tumors grew to a mean size of 835 mm^3^ (spheroid) and 850 mm^3^ (monolayer) after twenty weeks (Fig. 4a,c). The spheroid tumors ranged in size from 5.82 mm^3^ to 2041 mm^3^, while the monolayer tumors ranged from 7.71 mm^3^ to 2395 mm^3^. The initial tumors in the spheroid group were 5 mm^3^ to 10 mm^3^ in size. Two of these tumors grew rapidly, reaching approximately 700 mm^3^ and 2041 mm^3^ after only fourteen weeks (Fig. 4c). Four weeks later, the third early detected tumor had a tumor size greater than 700 mm^3^.

The earliest monolayer tumors were detected at six weeks with tumor volumes of 8 mm^3^ and 13 mm^3^ and at ten weeks with a size of 9 mm^3^. These tumors grew slower than spheroid tumors and reached a size of 750 mm^3^ and 220 mm^3^ after twenty weeks. The third tumor, with an initial volume of 9 mm^3^, proliferated rapidly from a volume of approximately 30 mm^3^ at week fourteen to approximately 1000 mm^3^ two weeks later. This tumor progressed to become the largest one, measuring 2395 mm^3^ (Fig. 4c).

After fourteen weeks, one mouse in each group developed a prostate tumor with a volume of approximately 10 mm^3^. However, these tumors increased to only 14 mm^3^ and 35 mm^3^ at week 20.

PSA levels in spheroid tumor bearing mice ranged from 0.11 ng/mL to 238.29 ng/mL, while PSA levels in monolayer tumor bearing mice ranged from 0.27 ng/mL to 260 ng/mL. At week fourteen, mean PSA levels were higher after intraprostatic spheroid implantation (88.75 ng/mL) than after single cell injection (23.15 ng/mL, p = 0.62) (Fig. 4b). The highest PSA level in spheroid tumors (238 ng/mL) occurred at fourteen weeks, while the highest PSA level in monolayer tumors (260 ng/mL) was measured at twenty weeks (Fig. 4d). The sudden decrease in tumor volume and PSA levels at week fourteen in the spheroid mice was due to the premature sacrifice of two mice with the largest tumors to that point (humane end point), as illustrated in the individual PSA and tumor volume development (Fig. 4c,d).

The tumor volume of LNCaP spheroids doubled on average after 10.5 days, while monolayer prostate tumors showed a doubling of tumor volume after 19.5 days (p = 0.1) (Fig. 4f). PSA doubling time averaged 9 days in spheroid mice compared to 16 days in monolayer mice (p = 0.4) (Fig. 4f).

Linear correlation analysis revealed a significant positive correlation between sonographic tumor volume and PSA (r^2^ = 0.82 and r^2^ = 0.74, respectively, and p < 0.0001) validating serum PSA as a valid non-invasive measure of tumor burden in our orthotopic model (Fig. 4e).

### Histological und immunohistochemical evaluation

**Figure 5:**
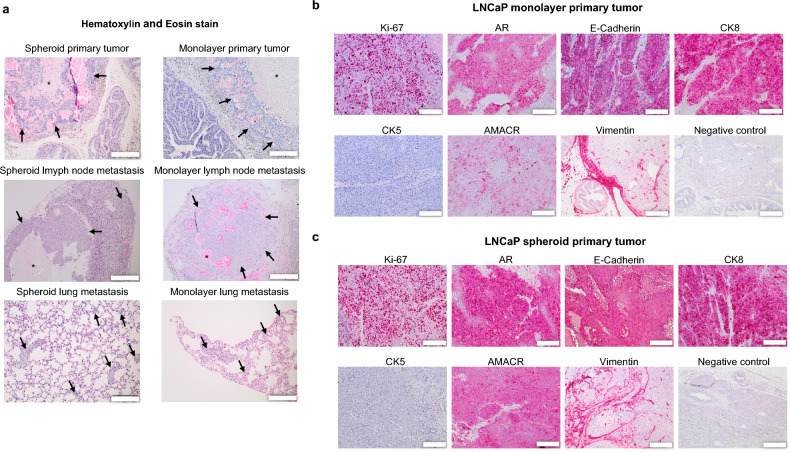
Histology and immunohistochemistry. (**a**) Histological evaluation (H&E staining) of spheroid and monolayer primary tumors and metastases. Tumors and metastases are labeled with arrows. Central necroses is labeled with asterisks (*). Scale bar = 200 μm. (**b**,**c**) Immunohistochemical expression of Ki-67, AR, E-Cadherin, CK8, CK5, AMACR and Vimentin in LNCaP monolayer and spheroid primary tumors*.* Scale bar = 200 μm.

**Figure 6:**
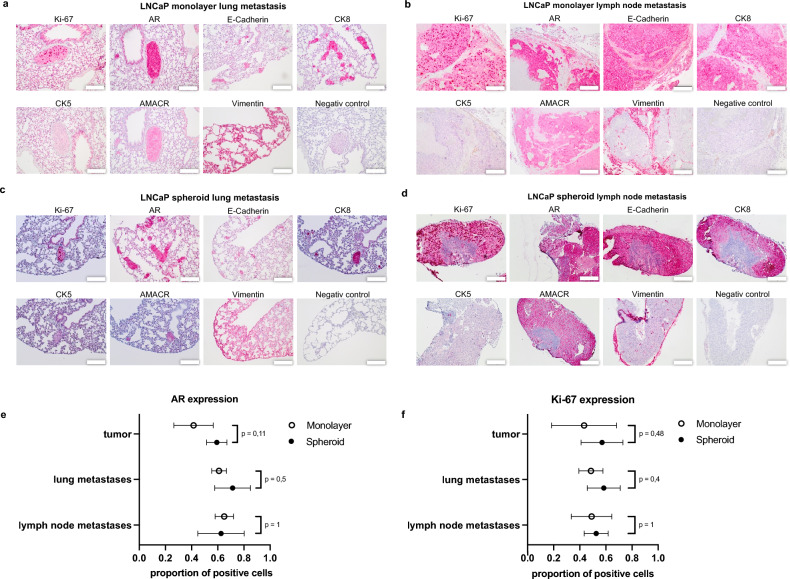
Immunohistochemistry of metastases and quantification of AR and Ki-67 expression in primary tumor and metastases. Immunohistochemical expression of Ki-67, AR, E-Cadherin, CK8, CK5, AMACR and Vimentin in LNCaP monolayer lung metastases (**a**), monolayer lymph node metastases (**b**), spheroid lung metastases (**c**) and spheroid lymph node metastases (**d**)*.* Scale bar = 200 μm. (**e**) Proportion of LNCaP cells with nuclear AR expression in primary tumors, lung and lymph node metastases of monolayer and spheroid tumors (mean with SD). (**f**) Proportion of Ki-67 expressing LNCaP cells in primary tumors, lung and lymph node metastases of monolayer and spheroid tumors (mean with SD).

Histological evaluation of the primary tumors revealed a solid tumor mass with central areas of necrosis and intratumoral hemorrhages (Fig. 5a). The pleomorphic tumor cells exhibited enlarged prominent nucleoli, a high nuclear-to-cytoplasmic ratio and numerous mitotic figures. In the tumor periphery, infiltrative tumor growth was observed, and a pseudofibrotic capsule was formed in some cases. In smaller primary tumors, the mouse prostate glands were often visible, whereas in larger tumors, they were completely displaced. Lung metastases presented as multiple tumor cell nests consisting of a few cells, some of which organized into tubular or spherical structures. The largest pulmonary metastases were approximately 200 μm in diameter (Fig. 6a) consisting of large pleomorphic tumor cells with prominent nuclei. The lymph node metastases were characterized by a solid cohesive tumor mass and necrotic zones. The histoarchitecture of the cortex, paracortex, and medulla remained well differentiated in most cases, and the lymph node capsule with marginal sinus was intact. Tumor cells reflected again the histological features of the primary tumors.

The primary tumors exhibited nuclear staining for Ki-67 and the androgen receptor (Fig. 5b and c). Androgen receptor expression was also visible in the cytoplasm. Additionally, the characteristic expression of cytokeratin 8 as luminal epithelial marker was identified, while CK5 was negative (Fig. 5b,c). Moreover, strong cytoplasmic expression of AMACR and E-cadherin was observed (Fig. 5b,c). The lung and lymph node metastases displayed the same marker expressions as the primary tumors (Fig. 6).

The proportion of tumor cells expressing nuclear AR was higher in spheroid primary tumors (over 60%) than in monolayer primary tumors (45%, p = 0.11) (Fig. 6e). In lung metastases of monolayer tumors, 60% of the tumor cells showed strong nuclear staining of the AR (Fig. 6e) in contrast to 70% in lung metastases from spheroid tumors (p = 0.5) (Fig. 6e). Lymph node metastases of spheroid tumors exhibited approximately 65% AR-positive cells, which was similar to the monolayer lymph node metastases (Fig. 6e).

The expression of the proliferation marker Ki-67 in spheroids was more pronounced in the peripheral proliferation zone. The expression in spheroid primary tumors was on average 20% higher than in monolayer primary tumors (p = 0.48) (Fig. 6f). The proportion of positive nuclear staining was more pronounced in lung metastases of the spheroids (60%) compared to monolayer lung metastases (50%, p = 0.4) (Fig. 6f). In lymph node metastases, around 50% of the tumor cells in both groups expressed Ki-67 (Fig. 6f).

## Discussion

This study describes the first successful orthotopic implantation of preformed 3D prostate cancer spheroids cells. Overall, there are few publications on the characterization of LNCaP spheroids. Specifically, no studies have been conducted on the long-term viability and growth of LNCaP spheroids of varying sizes. Therefore, these findings are—besides the establishment of the orthotopic implantation technique—an important contribution to the understanding of LNCaP spheroids and the effect of three-dimensionality of implanted PCa cells tumor growth in xenograft models.

Ohya et al. demonstrated the development of spheroids from single cells in ultra-low attachment well plates after 5–7 days, as in our study. They described a similar size of approximately 500 μm after 7 days when culturing 100,000 cells^16^. Ballangrud et al. and Watanabe et al. distinguished spheroids into three zones^17,18^. A central necrosis, a transition zone and an outer proliferation zone developed in spheroids larger than 500 μm. In the proliferation zone, tumor cells expressed increased levels of Ki-67, as observed in this study. In contrast to our findings, Jouberton et al. determined a shorter doubling time of approximately 33 h for LNCaP spheroids containing 1000 cells^19^. However, their monolayer LNCaP cell doubling time (19 h) a priori differed to our doubling time (60 h). As mentioned above, LNCaP spheroids form necrotic cores from about 500 μm in size, constituting about half of the spheroids^18,19^. Accordingly, a similar ratio should be expected for the proportion of viable cells to the total number of cells. The viability of 50% to 80% determined in this study supports this hypothesis. Song et. al. also illustrated a decreasing gradient of Ki-67 positive cells within a spheroid and about 70% Ki-67 positive LNCaP cells^20^. All in all, markers typical of prostate cancer were present both in spheroids and organ specimens. Except for Ki-67 and AR, no differential expression was observed between the groups.

The size of the mouse prostate and the diameter of the cannula used to inject were limiting factors for intraprostatic implantation of spheroids. To preserve the prostate's integrity, we used an injection cannula size of 20 G. We selected day 6 spheroids with an initial cell count of 25,000 LNCaP cells for orthotopic LNCaP spheroid transplantation as these spheroids exhibited a solid spheroidal shape, a high average cell viability and maintained their integrity when aspirated and injected with a 20 G needle. After six days, spheroids with an initial number of 25,000 LNCaP cells reached an average cell count of 40,000 cells. Consequently, 40,000 LNCaP cells were inoculated orthotopically as single cells from monolayer culture.

Spheroid aspiration was easier at low viscosity. Thus, a low concentration of cell culture medium to matrigel was chosen at a ratio of 1:1, lower than the matrigel concentration used in previously published orthotopic xenografts (1:3)^13^.

The achieved engraftment rate of 50% is very positive considering the low Matrigel concentration and the very low number of LNCaP cells. Recent publications on orthotopic transplantation of prostate cancer cells have reported growth rates of up to 90%, but with up to 20 times higher cell counts, ranging from 50,000 to 500,000 cells^14,21^. The transplantation of larger spheroids could potentially be achieved by embedding LNCaP spheroids in a solid carrier and then transplanting the plug under the prostate capsule. This would allow implantation of either one large spheroid containing for instance 100,000 cells or multiple smaller spheroids consisting of less cell numbers. Considering this aspect or by increasing the sample size in future studies we could improve the statistical power of our studies.

The tumor growth dynamics were confirmed by PSA measurements. The relevance of PSA as a progression marker of prostate cancer is supported by the strong positive correlation of tumor volume with PSA, as described in recent studies^14,21^. Interestingly, the local growth of prostate cancer in LNCaP spheroids was faster than after single cell injections as evidenced by the shorter tumor and PSA doubling times of LNCaP spheroids compared to monolayer tumors. The faster solid tumor development and the formation of larger tumors from LNCaP spheroids in a shorter period may be a result of earlier spheroid establishment, leading to an earlier proliferation. An additional intriguing observation is the higher rate of metastases in LNCaP spheroid mice (75%) compared to LNCaP single cell suspension mice (50%), although this observation has to be interpreted with caution due to the relatively low number of mice and lack of statistical significance. The more rapid development of tumors in the spheroid group and the higher rate of metastases may also be related to a higher proliferation of spheroid tumors, which is consistent with the increased proliferation indices in immunohistochemistry in spheroid tumors and metastases compared to monolayer specimens. Furthermore, the higher AR expression in spheroid tumor cells indicates an impact of the specific microenvironment of a spheroid on AR gene expression, although previous in vitro comparisons demonstrated invariant AR expression between monolayer and spheroid LNCaP cultures^22^. Elevated AR abundance possibly facilitates heightened tumor progression and metastasis in spheroid tumors. However, the upregulation of AR and Ki-67 in spheroid tumors does not achieve statistical significance, limiting the interpretation of the results.

Moreover, the proliferative advantage of spheroids could possibly be driven by AR- splice variants, such as AR-V7, which elevate the expression of AR responsive genes promoting tumor initiation and progression by ligand-independent AR pathway activation^23^.

Extracellular vesicles (EVs) possibly adopt a role in enhancing tumor proliferation^24,25^. In a study by Ramteke and colleagues, EV secreted by LNCaP cells under hypoxic conditions increased invasiveness and proliferation and decreased apoptosis in nearby tumor cells^26^. Given the characteristics of a spheroid, consisting of intense cell–cell contacts in a three-dimensional construct and a pronounced central hypoxia, these observations indeed suggest a rationale for the strong proliferation in spheroid tumors in vivo. In addition, tumor biology is strongly influenced by EVs from tumor-associated cells such as adipocytes, endothelial cells, immune cells, and fibroblasts^24,27,28^. Recently, Neuwirt et. al identified a tumor-associated fibroblast (CAFs) induced increase in androgen resistance^29^. Thus, to further investigate the tumor microenvironment (TME), heterospheroids consisting of LNCaP cells and CAFs, rather than homospheroids consisting of LNCaP cells alone, may provide a deeper understanding of the complex TME. In a previous study Linxweiler et. al coinoculated CAFs or non-cancer fibroblasts with prostate cancer cells (LuCaP136 spheroids or LNCaP cells). CAFs significantly stimulated primary tumor growth and metastatic spread in LuCaP136, while tumor growth in LNCaP cells was stimulated equally by all fibroblasts, suggesting a differential impact of the TME depending on the three-dimensionality of cancer cells^13^.

**Figure 7:**
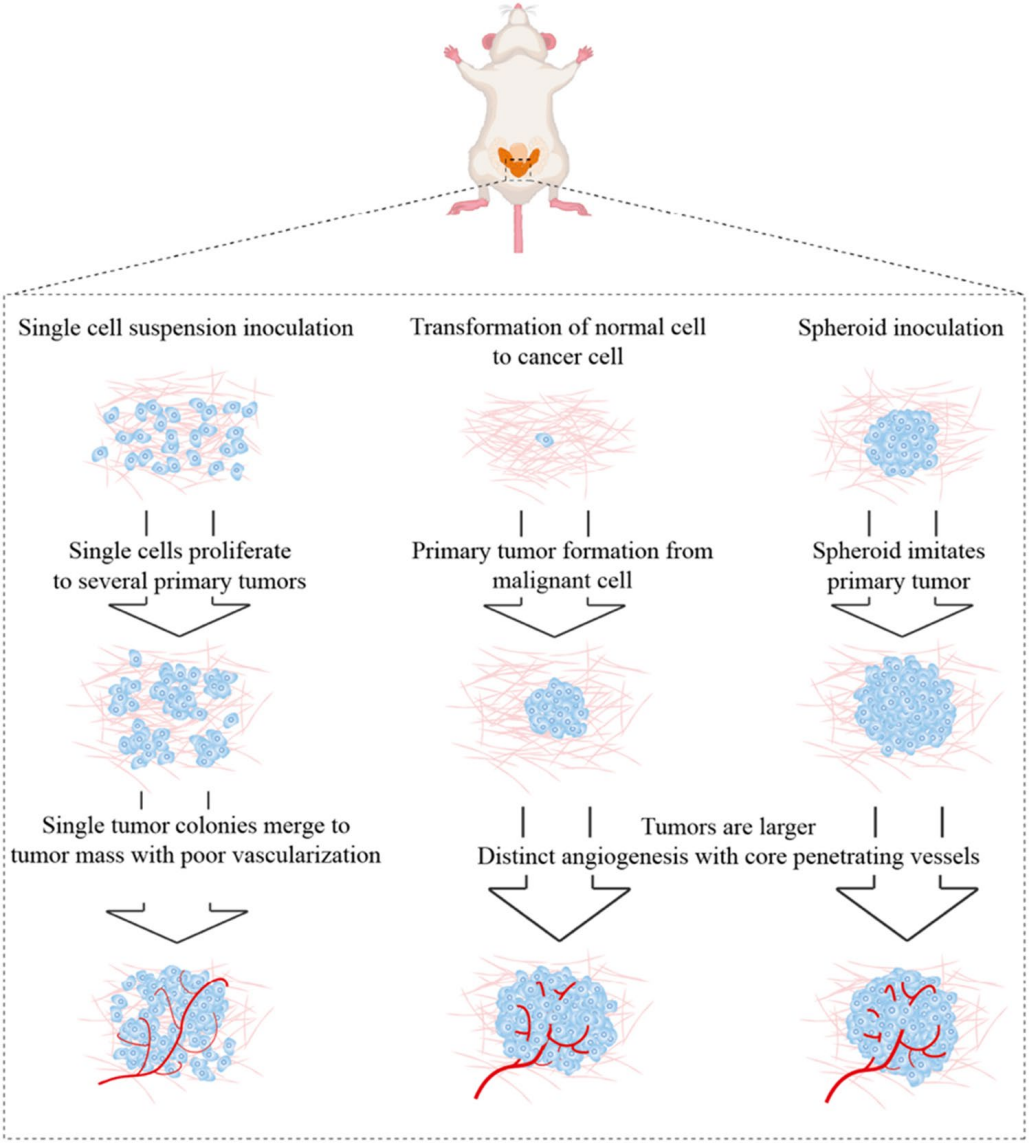
Schematic illustration of a hypothetical rationale for an accelerated tumor progression and metastasis in LNCaP spheroids compared to LNCaP single cells in relation to natural tumor development. Modified from Szade et al.^30^.

Another rationale for the enhanced proliferation and metastases of spheroid tumors is the increased vascularization of spheroid tumors (Fig. 7). Szade et. al. recently described increased vascularization of subcutaneously injected spheroid tumors of B16 melanoma and Lewis lung carcinoma compared to conventional single cell suspensions^30^. Neoangiogenesis in spheroid tumors extended into the tumor core, in contrast to peripheral vascularization in monolayer tumors. This could possibly facilitate the infiltration of cells promoting tumor proliferation^31,32^. A further hypothesis for accelerated tumor development in LNCaP spheroids involves the preformation of the spheroid in vitro. Conventional implantation of LNCaP cells in a single cell suspension from monolayer culture results in smaller tumor colonies that coalesce over time to form a solid tumor mass^30^. However, transplanting an assembled tumor mass as a spheroid suggests a continuous proliferation from one primary tumor lesion, similar to natural cancer development from one or few normal cells^33^. The formation of a single cell suspension to a tumor mass is delayed, as opposed to spheroids.

Therefore, the orthotopic implantation of spheroids presents a superior model for mirroring the complexity of human cancer in contrast to conventional monolayer single cell injections, providing a more physiological microenvironment. This study highlights the importance of considering the microenvironmental context when studying cancer progression. Nonetheless, the implications for human prostate cancer are limited by the metastatic origin of LNCaP cells and the absence of crucial microenvironment elements, such as fibroblasts and immune cells^34^.

The established model of spheroid implantation provides a fundamental platform for further mechanistic studies, elucidating the intricate molecular and cellular processes involved in the tumor-promoting effects of spheroids. To address these effects, key signaling pathways, cellular interactions, and changes in gene expression profiles should be examined.

The orthotopic spheroid implantation model presents distinct advantages compared to other common models, such as PDX, due to its high rates of engraftment and metastases^35^. Additionally, the formation of heterospheroids with precisely controllable cellular composition could provide a more accurate representation of a real tumor in future research. The ability to manipulate individual cellular components, such as up- or down-regulating genes or knocking them out with CRISPR/Cas, enables the investigation of the impact of specific cellular components on the microenvironment in the orthotopic spheroid model.

Previous in vitro studies have shown that spheroids exhibit greater resistance to chemo- and radiotherapy compared to conventional monolayer cultures^36,37^. Consequently, our model may suggest new therapeutic strategies or prompt a re-evaluation of therapeutic in vivo studies using conventional single cells from monolayer cultures. Given the tumor-promoting advantages of spheroids in vivo, this model could potentially be the preferred in vivo method for future prostate cancer research.

## Methods

### Animals

The in vivo experiments were performed with immunodeficient CB17-SCID male mice (6–8 weeks old) from Charles River Laboratories (Sulzfeld, Germany). The mice were kept under controlled temperature and humidity conditions with 12 h of ambient lighting in ventilated and pathogen-free cages in the animal facility of the Institute for Clinical Experimental Surgery at Saarland University. The animals had free access to drinking water and food and their health status was checked daily. All experiments were approved by the Ethical Review Board of Saarland (Reference Nr. 26/2020) and conducted in accordance with the German legislation on protection of animals and the National Institutes of Health Guide for the Care and Use of Laboratory Animals. The study is in accordance with the ARRIVE guidelines.

### Cell culture

LNCaP prostate cancer cells were cultivated as monolayer cultures in RPMI medium (Sigma-Aldrich, St. Louis) supplemented with 10% fetal bovine serum (Sigma-Aldrich) at 37 °C, 95% humidity and 5% CO_2_. LNCaP spheroids were generated by seeding of LNCaP cells (5,000–25,000 per well) in ultra-low attachment 96-well plates (Corning, New York). Medium was replaced every 48 h. The cell count and viability of spheroids was determined with Luna II Automated Cell Counter (Logos Biosystems) after dissociating spheroids with a 100 µl pipette. For orthotopic injection of single cell suspensions, the cells were harvested, counted, and suspended in a 1:1 mixture of Matrigel HC (Corning) and RPMI culture medium at a density of 40,000 cells/10 μl and preserved on ice until injection. Immediately prior to intraprostatic injection, the suspension was redissolved and aspirated air-free into a cooled 10 μl Hamilton syringe (Hamilton, Reno, NV). Spheroids were prepared for orthotopic implantation in one well of a 96 well ultra-low attachment plate in a 100 µl 1:1 Matrigel to medium suspension. Using a 1 ml syringe and a cooled 20-gauge cannula, the six-day maturated spheroid (starting cell number: 25,000) was aspirated.

### Orthotopic tumor cell/spheroid injection and follow-up

**Figure 8:**
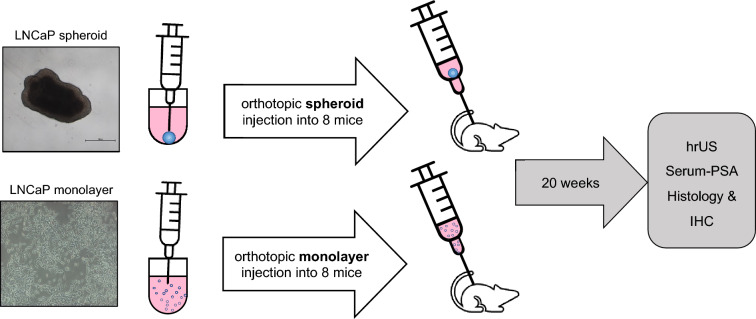
Schematic illustration of the in vivo experiment setup and follow-up. Eight mice were implanted orthotopically with one LNCaP spheroid each consisting of 40,000 LNCaP cells. Another eight mice in the control group received orthotopic injection of 40,000 LNCaP cells as single cell suspension. The follow-up period lasted 20 weeks and included regular ultrasonography and PSA measurements. After autopsy, the dissected organs were evaluated by histology and immunohistochemistry. hrUS = high-resolution 3D ultrasonography; IHC = immunohistochemistry.

Intraprostatic tumor cell implantation was implemented with intraperitoneal anesthesia applying 75 mg/kg ketamine and 15 mg/kg xylazine under stereo microscopy (Leica M651; Leica Microsystems AG, Heerbrugg, Switzerland). After fixation in a supine position, the lower abdominal skin was median incised over 10 mm. A median laparotomy was used to expose the bladder, the seminal vesicles and the anterior lobes of the prostate. Then, 10 µl of the 1:1 Matrigel medium suspension containing 40,000 LNCaP cells was injected into the left anterior prostate lobe utilizing a cooled Hamilton syringe (Fig. 8). The prostate capsule was perforated just below the left vas deferens, the syringe was inserted 5–6 mm laterally into the anterior prostate lobe, the tumor cell suspension was slowly injected and a reflux was prevented by applying gentle pressure with a moistened cotton swab.

Spheroids in 1:1 Matrigel medium suspension were aspirated with a 1 ml syringe and a cooled 20-gauge cannula and subsequently, as specified above, 10–30 µl of the suspension containing a single, preformed LNCaP spheroid were injected into the left anterior prostate lobe. To confirm the preserved integrity of spheroids after injection, we assessed the morphology of spheroids by microscopy before and after aspiration and injection with a 20-gauge needle. As with the injection of single cells, reflux was prevented by applying gentle pressure with a moistened cotton swab to the injection site. The muscle and skin layers were then closed separately with a fast-absorbing 5/0 Vicryl-rapid suture. To alleviate pain, the mice were treated with a single dose of 4 mg/kg carprofen subcutaneously after surgery and 20 mg/kg tramadol via drinking water from the first pre-operative day until the third post-operative day. To monitor tumor growth, high-resolution 3D ultrasonography was performed on all mice every two weeks as of week four and blood samples were obtained for PSA analyses as of week two. At the end of the 20 weeks follow-up period, the mice were cervically dislocated under anesthesia and at autopsy, the prostate tumor, lungs, a femur bone, liver, aortic lymph nodes, brain and spleen were retrieved for histological and immunohistochemical analysis (Fig. 8).

### High resolution 3D ultrasonography (hrUS) and serum PSA measurements

HrUS of the primary tumors was performed with a Vevo 2100 Small Animal Ultrasonography System (FUJIFILM Visual Sonics, Toronto, Canada). After anesthesia induction with isoflurane (4%, flow rate of 400 ml/min) and establishment of complete sedation, the mice were fixed in supine position on a heated stage and anesthesia was continued via mask (2% isoflurane; flow rate 200 ml/min). The abdomen was then shaved and a depilatory cream was applied. After residual hair had been removed, the warmed contact gel (Aquasonic, Parker, Fairfield, NJ) was applied generously in apricot size. Once the prostate was centered in the focus of the MS400 transducer, the 3D acquisition could be started. A motorized mechanism traversed the ultrasound transducer across the abdomen, generating 2D images of the prostate region in a transversal orientation at regular 200 µm intervals over a length of 2 cm. Once the images were generated, isoflurane flow was halted, and the mouse removed from the device. In the cross-sectional 2D images, the perimeter of the tumor was marked for tumor volume calculation via the volumetric analysis function of the ultrasound software (Vevo LAB Software, FUJIFILM Visual Sonics, Toronto, Canada).

While sedated, a venous blood sample (~ 50 µl) was collected using a glass microcapillary through puncture of the retroorbital venous complex (Sigma-Aldrich). After centrifugation (10 min, 6000 rpm), the serum samples were analyzed in the laboratories of the Saarland University Medical Center using an Electro-chemiluminescence immunoassay (ECLIA) in a COBAS 1 immunoassay system (Roche, Basel, Switzerland).

### Histology an immunohistochemistry

In preparation for histology and immunohistochemistry, spheroids were embedded in agarose gel cylinders (Sigma-Aldrich, St. Louis, USA) at the bottom of 1.5 ml Eppendorf tubes (Eppendorf, Hamburg, Germany). Agarose tubes with spheroids, as well as dissected organs, were formalin-fixed and paraffin-embedded using standard protocols. A rotation microtome (Leica RM 2125RT; Leica microsystems, Wetzlar, Germany) was used to mount 4 µm thin sections on glass slides. H&E staining was performed using standard protocols. Immunohistochemistry was conducted using a Dako REAL™ labeled streptavidin–biotin detection system (Agilent Technologies, Santa Clara, CA) as previously described^38^. Antibodies against androgen receptor (AR; Sigma-Aldrich, St. Louis, MO), Ki67 (Dako, Glostrup, Denmark), alpha-methyl-CoA-racemase (AMACR; Invitrogen, Waltham, MA), cytokeratin 8 (CK8; Sigma-Aldrich), cytokeratin 5 (CK5; Diagnostic BioSystems, Pleasanton, CA), vimentin (Cell Signaling, Danvers, MA) and E-cadherin (Invitrogen) were used. The epitopes of the androgen receptor, CK8, and AMACR were unmasked using citrate buffer, while the epitopes of Ki-67, CK5, vimentin, and E-cadherin were unmasked employing Tris–EDTA buffer. Antibody dilution varied between staining of mouse organs and spheroids, as illustrated in the supplements (supplement table S1).

The quantitative analysis of immunohistochemistry images was performed with the ImageJ/FIJI plugin “colour deconvolution 2” from Gabriel Landini^39^, based on Ruifrok and Johnston^40^. The analysis was implemented with the protocols from Jie Shu^41^.

### Statistical analyses

The statistical analyses were performed with IBM SPSS Statistics 27 (Armonk, NY). Generally, tow-tailed Mann–Whitney-U-tests were employed to evaluate statistical significance whilst assuming a significance level of p < 0.05. The graphics were generated with GraphPad Prism 9 (Boston, MA) and the schematic illustrations were created with Microsoft® PowerPoint (version 16.73; Redmond, WA) and Adobe Acrobat Illustrator (version 27.9.0; San José, CA).

### Supplementary Information

Supplementary Information.

## Data Availability

The datasets generated during the current study are available from the corresponding author on reasonable request.
