# Supplementary material for: Tumor promoting effect of spheroids in an orthotopic prostate cancer mouse model
Source: Sci Rep. 2024 Apr 17;14:8835. doi: 10.1038/s41598-024-59052-0 (PMC11024136; doi:10.1038/s41598-024-59052-0)
Supplement: Supplementary file 1 — Supplementary Information. [file 41598_2024_59052_MOESM1_ESM.docx]

**Supplementary information**

**Tumor promoting effect of spheroids in an orthotopic prostate cancer mouse model**

Authors:

Julius Lars Daniel Bastian^1^, Philip Zeuschner^1^, Michael Stöckle^1^, Kerstin Junker^1^, Johannes Linxweiler *^1^

Authors´ affiliations:

^1^Saarland University, Department of Urology and Pediatric Urology, Homburg/Saar, Germany

**Suppl. Table S1: Antibody dilution**

| Antibody | Name | Manufacturer and order number | Dilution spheroids | Dilution primary tumors | Dilution lymph node metastases | Dilution lung metastases |
| --- | --- | --- | --- | --- | --- | --- |
| AR | Polyclonal Rabbit Anti-Androgen Receptor Antibody | Sigma-Aldrich  A9853 | 1:2000 | 1:2000 | 1:4000 | 1:4000 |
| AMACR | Polyclonal Rabbit Anti Alpha-Methylacyl-CoA racemase  Antibody | Invitrogen  PA5-23619 | 1:500 | 1:200 | 1:1000 | 1:500 |
| CK 5 | Monoclonal Mouse Anti- Cytokeratin 5 Antibody | Diagnostik Biosystems  MOB361  *Klon XM26* | 1:50 | 1:50 | 1:50 | 1:50 |
| CK 8 | Monoclonal Mouse Anti-Cytokeratin Peptide 8 Antibody | Sigma-Aldrich  C5301  *Klon M20* | 1:200 | 1:800 | 1:800 | 1:800 |
| E- Cadherin | Monoclonal Mouse Anti- E-Cadherin Antibody (HECD-1) | Invitrogen  13-1700 | 1:800 | 1:1600 | 1:1600 | 1:3000 |
| Ki- 67 | Monoclonal Mouse Anti-Ki-67  Antibody | Dako  M7240  *Klon MIB-1* | 1:100 | 1:100 | 1:100 | 1:100 |
| Vimentin | Monoclonal Rabbit Vimentin (D21H3) XP Antibody | Cell Signaling  5741 | 1:100 | 1:100 | 1:100 | 1:100 |
